# Model of the Mediator middle module based on protein cross-linking

**DOI:** 10.1093/nar/gkt704

**Published:** 2013-08-10

**Authors:** Laurent Larivière, Clemens Plaschka, Martin Seizl, Evgeniy V. Petrotchenko, Larissa Wenzeck, Christoph H. Borchers, Patrick Cramer

**Affiliations:** ^1^Gene Center Munich and Department of Biochemistry, Center for Integrated Protein Science Munich (CIPSM), Ludwig-Maximilians-Universität München, Feodor-Lynen-Str. 25, 81377 Munich, Germany and ^2^Department of Biochemistry and Microbiology, Genome British Columbia Protein Centre, University of Victoria, No. 3101-4464 Markham Street, Vancouver Island Technology Park, Victoria, BC V8Z7X8, Canada

## Abstract

The essential core of the transcription coactivator Mediator consists of two conserved multiprotein modules, the head and middle modules. Whereas the structure of the head module is known, the structure of the middle module is lacking. Here we report a 3D model of a 6-subunit Mediator middle module. The model was obtained by arranging crystal structures and homology models of parts of the module based on lysine–lysine cross-links obtained by mass spectrometric analysis. The model contains a central tetramer formed by the heterodimers Med4/Med9 and Med7/Med21. The Med7/Med21 heterodimer is flanked by subunits Med10 and Med31. The model is highly extended, suggests that the middle module is flexible and contributes to a molecular basis for detailed structure–function studies of RNA polymerase II regulation.

## INTRODUCTION

Mediator is a central and conserved coactivator complex required for gene transcription by RNA polymerase (Pol) II (1–6). Mediator connects gene-specific transcription factors and the general Pol II machinery. Mediator from the yeast *Saccharomyces cerevisiae* has a molecular mass of 1.4 MDa and consists of 25 subunits that were assigned to four modules called head, middle, tail and kinase modules. The head and middle modules constitute the functional core of Mediator (7). The Mediator core subunits are conserved throughout eukaryotes (8). The crystal structure of the 7-subunit Mediator head module has been solved at 4.3-Å resolution for *S. cerevisiae* (9,10) and at 3.4-Å resolution for *Schizosaccharomyces pombe* (11).

The structure of the middle module remains unknown. The *S. cerevisiae* middle module comprises four essential subunits, Med4, Med7, Med10 (Nut2) and Med21 (Srb7), and three nonessential subunits, Med1, Med9 (Cse2) and Med31 (Soh1). Detailed structural information on parts of the middle module is limited to two subcomplexes, the heterodimers Med7N/Med31 (12) and Med7C/Med21 (13), where Med7N and Med7C correspond to the N- and C-terminal regions of Med7, respectively. We previously reported the expression and purification of a recombinant 7-subunit Mediator middle module (14), and found that the high intrinsic flexibility of the module prevents its crystallization.

To investigate the 3D subunit architecture of the middle module, we report here a new protocol for the heterologous expression and purification of a 6-subunit middle module lacking subunit Med1. We subjected the purified middle module to chemical lysine–lysine cross-linking and identified pairs of cross-linked sites by mass spectrometry (CX-MS). CX-MS is a novel and powerful method for obtaining the subunit architecture of large protein complexes (15). We previously applied CX-MS to multiprotein complexes involved in transcription (16–18). By combining the cross-linking information with known structures and structure-based homology modeling, we derived an architectural model of the Mediator middle module that provides the relative orientation of subunits and guides future structural and mechanistic studies of Mediator function.

## MATERIALS AND METHODS

### Preparation of a 6-subunit Mediator middle module

**Figure 1.**
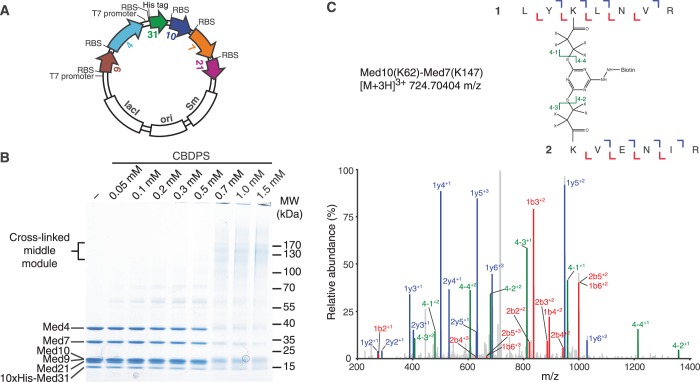
Preparation and CX-MS analysis of the Mediator middle module. (**A**) Schematic representation of the plasmid used for Mediator middle module recombinant expression. Coding sequences are colored according to a code used throughout (Med4, cyan; Med7, orange; Med9, brown; Med10, slate; Med21, magenta; Med31, green). Co-expression was driven from a single plasmid with two T7 promoters, one for bicistronic expression of Med9 and Med4, and one for tetracistronic expression of Med31, Med10, Med7 and Med21. His tag, deca-histidine tag; *ori*, origin of replication; *lacI*, gene encoding Lac repressor; RBS, ribosome binding site; *Sm,* streptomycin resistance gene. (**B**) SDS-PAGE analysis of the middle module cross-linked with different concentrations of CBDPS. (**C**) Fragmentation spectrum of a cross-linked peptide.

Bacterial co-expression of the *S. cerevisiae* Mediator middle module was performed using a single plasmid based on a pCDFDuet-1 vector (Novagen), shown schematically in Figure 1A. Open reading frames were cloned sequentially and additional ribosomal binding sites were introduced as described (13). Med31 harbors a deca-histidine tag at its N-terminus. The exact sequence of the construct is available on request. The middle module was expressed in *Escherichia coli* BL21 CodonPlus(DE3)RIL cells (Stratagene). Cells were grown in Luria broth medium at 37°C to an optical density of 0.5 at 600 nm. Expression was induced with 0.5 mM isopropyl-β-d-1-thiogalactopyranoside for 16 h at 18°C. Cells were lysed by sonication in buffer A [50 mM Tris pH 8.0, 150 mM sodium chloride, 5 mM dithiothreitol (DTT)] containing protease inhibitors (19). After centrifugation, the supernatant was loaded onto a 2-ml Ni-NTA agarose bead column (QIAGEN) equilibrated in buffer A. The column was washed with buffer A containing increasing concentrations of imidazole (0, 20, 50 mM). The complex was eluted with buffer A containing 300 mM imidazole. The middle module was further purified by anion exchange chromatography with a 1-ml HiTrap Q HP column (GE Healthcare). The column was equilibrated in buffer B (50 mM Tris pH 8.0, 50 mM sodium chloride, 2 mM DTT), and proteins were eluted with a linear gradient from 50 mM to 1 M sodium chloride in buffer B. Fractions containing middle module were applied to a HiLoad 16/600 Superdex 200-pg (GE Healthcare) exclusion column equilibrated in buffer C (20 mM HEPES-KOH pH 7.5, 150 mM potassium acetate, 10% (v/v) glycerol, 2 mM DTT). The protein complex was concentrated to 3 mg/ml, flash-frozen and stored at −80°C.

### Chemical protein cross-linking

The pure middle module was cross-linked using isotopically coded cyanurbiotindipropionyl succinimide (CBDPS, Creative Molecules Inc.) (20). The middle module was diluted to 0.5 mg/ml with buffer D (1× phosphate buffered saline, 2 mM DTT). CBDPS was dissolved in DMSO to 10 mM. To determine the optimal ratio of CBDPS to middle module, we mixed 3 µg of middle module with CBDPS at a concentration of 0.05–1.5 mM, and incubated for 30 min at 30°C. The reaction was stopped by addition of 0.5 M NH_4_HCO_3_ to a final concentration of 40 mM and incubation for 10 min at room temperature, and analyzed by sodium dodecyl sulfate–polyacrylamide gel electrophoresis (SDS–PAGE) (Figure 1B). The optimum concentration of CBDPS was considered to result in a higher molecular weight band. We used a final concentration of 0.7 mM CBDPS.

The cross-linked sample was dialyzed twice in dialysis buttons (Hampton Research) against 20 ml of buffer D. Trypsin and/or GluC were, respectively, added in a 1:10 or 1:1 ratio of protease to middle module and incubated overnight at 37°C. Proteases were then inhibited by addition of 10 mM 4-(2-aminoethyl)benzene-sulfonylfluoride and 20 mM phenylmethanesulfonylfluoride, and incubation for 10 min at room temperature. Affinity enrichment was performed with monomeric avidin beads (ThermoFisher) equilibrated with 0.1 M ammonium acetate. The amount of bead slurry was adjusted to a ratio of 1:10 of total CBDPS to bead capacity (1.2 mg/ml). The sample was loaded five times. The column was washed with 300 µl of ammonium acetate at concentrations of 0.1, 0.5 and 0.1 M, followed by three 300 µl water washes. The pH was adjusted to 2–3 by addition of 0.1% trifluoroacetic acid (TFA). Peptides were eluted with buffer containing 0.1% TFA and 50% acetonitrile. The sample was concentrated to 10 µl by lyophilization.

### Mass spectrometry analysis

Mass spectrometric analysis was carried out with a nano high performance liquid chromatography system (Easy-nLC II, ThermoFisher) coupled to the electrospray ionization (ESI) source of an LTQ Orbitrap Velos mass spectrometer (ThermoFisher Scientific). Samples were injected onto a 100 μm ID, 360 μm OD IntegraFrit trap column (New Objective Inc.) packed with Magic C_18_AQ (5 µm particle size, 100 Å pore size, Bruker-Michrom) and desalted by washing for 15 min at a flow rate of 300 nl/min with 0.1% (v/v) formic acid. Peptides were subsequently injected on a 75 μm ID, 360 μm OD IntegraFrit analytical column packed with Magic C_18_AQ (5 µm particle size, 100 Å pore size), equilibrated with 95% solvent A (2% (v/v) acetonitrile, 98% (v/v) water, 0.1% (v/v) formic acid) and 5% solvent B (90% (v/v) acetonitrile, 10% (v/v) water, 0.1% (v/v) formic acid). Peptides were separated at a flow rate of 300 nl/min using a 70 min gradient (0–60 min: 4–40% solvent B, 60–62 min: 40–80% solvent B, 62–70 min: 80% solvent B).

Mass spectromerty (MS) data were acquired with Xcalibur (version 2.1.0.1140) with mass tags and dynamic exclusion precursor selection methods enabled in global data-dependent settings. For CBDPS-H8/D8 mass difference between light and heavy isotopic forms of 8.05824 Da was used in mass tags setting. Mass tags and inclusion list runs used a Top 3 method. MS scans (m/z range from 400 to 2000) and MS/MS scans were acquired in the Orbitrap mass analyzer at resolutions of 60 000 and 30 000, respectively. Fragment ions for MS/MS acquisition were produced by collision-induced dissociation at normalized collision energy of 35% for 10 ms at activation *q* = 0.25. Data analysis was performed with DXMSMS match of ICC-CLASS (21). Two additional cross-link pairs [Med7(K35)–Med7(K103) and Med4(K36)–Med9(K117)] were obtained from a preliminary 7-subunit middle module preparation and were included in the final data set.

### Structure prediction and modeling

Protein sequences of Med4, Med9 and Med10 from fungal species (22) and higher eukaryotes were aligned with MUSCLE (23). The generated alignments were used for structure prediction with HHPred (24). Med4, Med9 and Med10 were predicted to be partly homologous to Med7, Med21 and Med8, respectively (Table 1).

**Table 1.** HHPred predictions for Med4, Med9 and Med10

Query subunit	Query residues	Template subunit	PDB code (chain ID)	Template residues	*P*-value	Score
Med4	46–127	Med7	1YKH (A)	19–108	0.0014	28.1
Med9	84–149	Med21	1YKH (B)	56–118	5.1 E-06	39.2
Med10	3–91	Med8	4H63 (H)	1–111	0.002	27.6

For middle module modeling, the *S. pombe* Mediator head structure (PDB code 4H63) (11) was used as a template. *Saccharomyces cerevisiae* Med11N/Med22N (PDB code 3R84) was superimposed on *S. pombe* Med11/Med22, either superimposing *S. cerevisiae* Med22N on *S. pombe* Med11 or superimposing *S. cerevisiae* Med22N on *S. pombe* Med22. Med7C/Med21 (PDB code 1YKH) (13) was then superimposed on *S. cerevisiae* Med11N/Med22N for both positions. Protein superimposition was performed using secondary structure matching in COOT (25). A second Med7C/Med21 dimer was positioned in the same relative orientation as in the crystal, letting the open ends of the C-terminal coiled coils to interact with each other (13). The two resulting models, formed of Med8 and two copies of Med7C/Med21, were used as templates for final middle module modeling using MODELLER (26). For this purpose, Med8 and the second copy of Med7C/21 served as templates for their structurally homologous regions in Med10 and Med4/9, respectively.

## RESULTS

### Improved expression and purification of recombinant Mediator middle module

Detailed structural analysis of the previously reported recombinant 7-subunit middle module (14) was hampered by the relatively low yields, nonstoichiometry of subunits and a tendency of the protein to aggregate. We found that omitting the Med1 subunit led to preparations of a 6-subunit middle module with improved biochemical properties. Our initial strategy to prepare the 6-subunit middle module relied on the separate expression of a 4-subunit subcomplex comprising Med7, Med10, Med21 and Med31 and of a 2-subunit subcomplex comprising Med4 and Med9, and their partial purification before *in vitro* assembly (14). Stoichiometric and highly soluble preparations were, however, only obtained when we co-expressed all six subunits from a single plasmid (Figure 1A). The complex was purified by affinity chromatography using a single deca-histidine tag located on the Med31 subunit, leading to a purified complex that contained all subunits in apparently stoichiometric amounts (Figure 1B, lane 1). Subsequent chromatographic analysis confirmed that the sample was monodisperse (data not shown).

### Cross-linking MS analysis of the Mediator middle module

**Figure 2.**
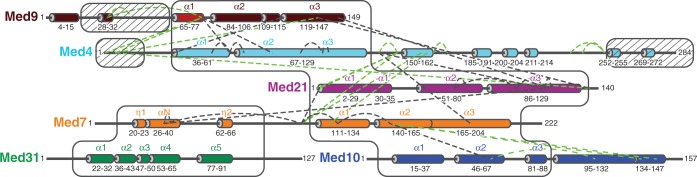
Map of lysine–lysine cross-link pairs of the Mediator middle module. The primary structure of the six subunits is drawn schematically to scale. *α*-Helices from crystal structures (12,13) or from predictions (27) are indicated as cylinders and colored as in Figure 1. *α*-Helices for which crystal structures are available or which could be modeled are labeled. White boxes enclose regions that could be modeled. Hatched boxes indicate regions that are dispensable for module assembly (14). Black and green dashed lines indicate cross-links that could be mapped onto the middle module model or not, respectively.

Fifty micrograms of middle module was incubated with isotope-labeled CBDPS (20). CBDPS reacts with primary amines in lysine side chains and protein N-termini, and harbors a biotin moiety. Cross-linking efficiency was monitored by SDS-PAGE (Figure 1B). After protease digestion, cross-linked peptides were enriched by affinity chromatography using avidin. Peptides and their fragments were detected with high-resolution liquid chromatography–MS (Methods). Measurements and subsequent analysis resulted in 55 mass spectra that matched cross-linked peptides (Supplementary Table S1). An example is shown in Figure 1C. These spectra correspond to 40 unique linkage pairs, among which 19 and 21 were intra- and inter-subunit cross-link pairs, respectively (Figure 2).

**Figure 3.**
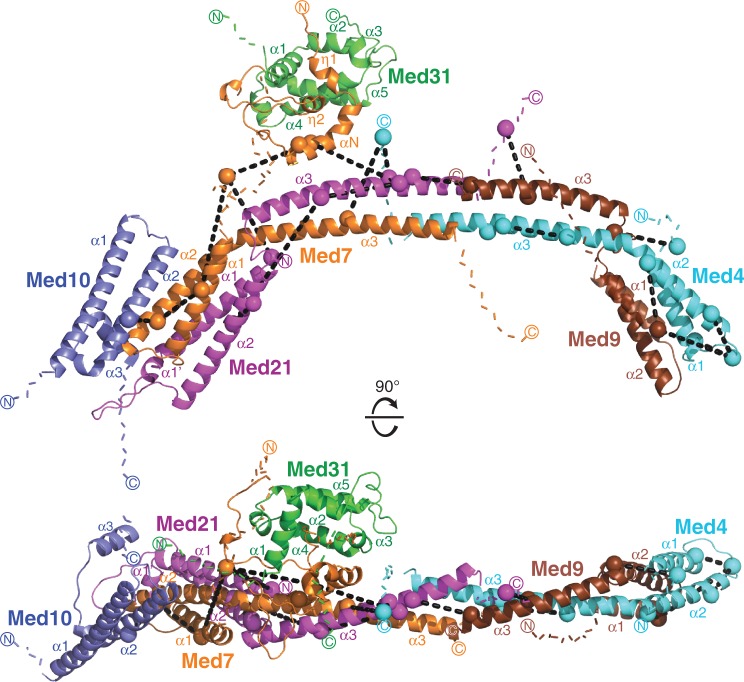
Model of the Mediator middle module. Subunits are shown as ribbon and colored as in Figure 1. Med9 helix *α*1 is represented as a semitransparent ribbon because its exact position remains unclear. The C*α* atoms of cross-linked residues are shown as spheres. Colored dashed lines indicate the first 10 residues extending from both termini of the model. Black dashed lines indicate inter-subunit or intra-subunit cross-links. The two views are related by a 90° rotation around a horizontal axis.

**Figure 4.**
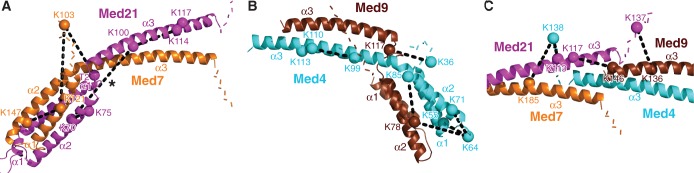
Close-up views of cross-links mapped on various regions of the model. (**A**) The Med7C/Med21 heterodimer structure. (**B**) Med4/Med9 heterodimer model. (**C**) Med4/Med9–Med7C/Med21 tetramer model interface. Subunits are shown as ribbon and colored as in Figure 1. The C*α* atoms of cross-linked residues are shown as spheres and labeled. Colored dashed lines indicate the first 10 residues extending from both termini of the model. Black dashed lines indicate inter-subunit or intra-subunit cross-links. A star indicates the intra-subunit crosslink in Med21, which did not fall within the acceptable distance cutoff.

The 40 cross-links that we obtained covered all proteins of the middle module except Med31 (Figure 2). Eight cross-link pairs could be mapped on the two known crystal structures of middle module subcomplexes, seven on the Med7C/Med21 subcomplex and one on the Med7N/Med31 subcomplex (Figures 2, 3 and 4A). The mapped cross-link pairs fell within the acceptable distance between Cα atoms of ≤26 Å, which corresponds to the length of the CBDPS spacer (14 Å) plus two times the length of a lysine side chain (6 Å). Only one intra-subunit cross-link in Med21 did not fall within the acceptable distance, possibly because of the flexibility of the protein. This analysis provided a positive control and demonstrated the reliability of our approach. We also performed cross-linking with a preparation of the complete 7-subunit middle module that additionally contained Med1. Owing to the worse biochemical behavior of the sample, however, fewer cross-links were obtained, and no additional cross-link pairs could be identified. We concluded that Med1 does not introduce major structural changes in the middle module, and continued our analysis with the 6-subunit module.

### Model of the Med4/Med9 heterodimer

**Figure 5.**
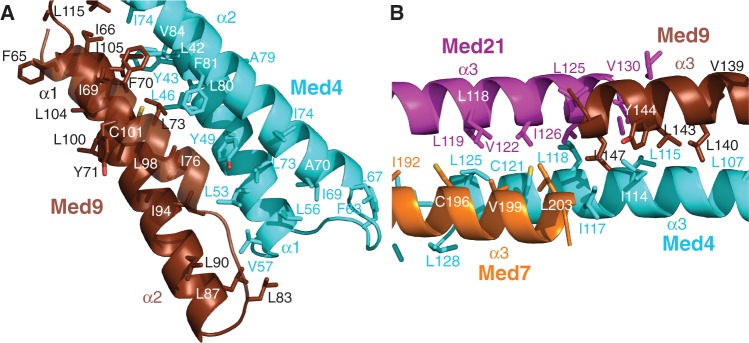
Close-up views of the Mediator middle module model. (**A**) Med4/Med9 4-helix bundle. (**B**) Med4/Med9–Med7/Med21 heterodimer–heterodimer interface. Subunits are shown as ribbons, with side chains of hydrophobic residues depicted as sticks. Carbon atoms are colored as in Figure 1; oxygen and sulfur atoms are colored red and yellow, respectively.

Crystal structures only cover 361 of the 1075 residues in the 6-subunit middle module, corresponding to 34%. Previously, we provided evidence that middle module subunits share the 4-helix bundle fold observed for the Med7C/Med21 and Med11N/Med22N heterodimers (19). In particular, parts of Med4 and Med9, which form a stable heterodimer (14), are predicted to be structurally similar to Med7C and Med21, respectively (Table 1). Based on this, we generated a homology model of the Med4/Med9 heterodimer (Figure 3). The model explained all seven cross-links observed within the Med4/Med9 heterodimer (3 intra-subunit cross-links, 4 inter-subunit cross-links, Figures 2, 3 and 4B). The model was further supported by the resulting location of hydrophobic residues, which form the interface between the two subunits (Figure 5A).

### Model of the Med4/Med9–Med7C/Med21 tetramer

To position the Med4/Med9 model relative to the Med7C/Med21 heterodimer crystal structure, we assumed that the two heterodimers interact as observed for symmetry-related Med7C/Med21 heterodimers in the Med7C/Med21 crystal structure (13). In both crystal forms of Med7C/Med21, heterodimers pack against each other via the conserved ends of their protruding coiled coils (13). The resulting Med4/Med9–Med7C/Med21 model is strongly supported by four cross-links observed between the two heterodimers (Figures 2, 3 and 4C). The model is further supported by a clustering of hydrophobic residues in the heterodimer–heterodimer interface (Figure 5B).

### Position of Med10 and Med31 on the module

**Figure 6.**
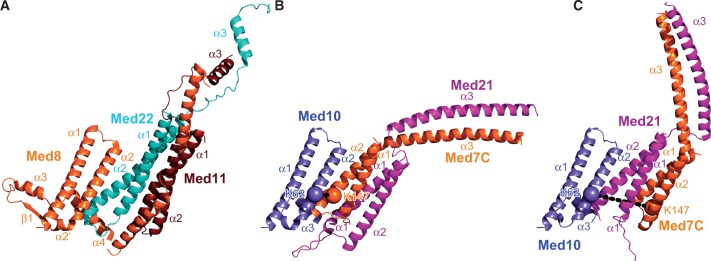
Possible models for the Med7C/Med10/Med21 trimer based on the Med8/Med11/Med22 trimer. (**A**) Med11/Med8/Med22 head subcomplex (PDB code 4H63). (**B**) Homologous middle subcomplex obtained by overlaying Med7C, Med10 and Med21 on Med22, Med8 and Med11, respectively. (**C**) Homologous middle subcomplex obtained by overlaying Med7C, Med10 and Med21 on Med11, Med8 and Med22, respectively. Black dashed lines indicate cross-link between Med10 residue K62 and Med7 residue K147.

Tertiary structure prediction of the Med10 subunit (24) suggests that Med10 is homologous to the N-terminal region of the head module subunit Med8, for which the structure is available (Table 1) (9–11). We modeled the corresponding Med10 region, which forms three helices, α1, α2 and α3, corresponding to helices α1, α2 and α4 in Med8 (11). In the head module structure, these three helices are part of the arm and spine, and interact with Med11N/Med22N (Figure 6A). In particular, Med8 helix α2 interacts with Med22 helix α2.

Because Med10 interacts with the Med7C/Med21 heterodimer (13,14), and because Med22 can be the structural homolog of either Med7 or Med21, Med10 may use its helix α2 to interact with either Med7 or Med21. Thus, two possible models result for a Med10–Med7C/Med21 trimeric subcomplex in the middle module (Figure 6B and C). However, only one of these two models, the one obtained by superimposing Med7C with Med22 (Figure 6B), is compatible with the observed cross-link between Med10 helix α2 and Med7C helix α2 (Figures 2 and 3). These data and considerations resulted in a reliable model of the core region of the middle module containing the bodies of five subunits.

Finally, two cross-links guided an approximate positioning of the external Med7N/Med31 subcomplex structure onto the obtained model. One cross-link was mapped between Med7N and a flexible linker in Med7C, and another one between Med7N and helix α3 in Med21. These restraints were not sufficient to ascribe a unique position to Med7N/Med31, but strongly suggest that this subcomplex is located near the Med7C/Med21 coiled coil (Figure 3). These results befit our previous notion that Med7N/Med31 forms a flexibly linked surface subcomplex on Mediator (12).

## DISCUSSION

**Figure 7.**
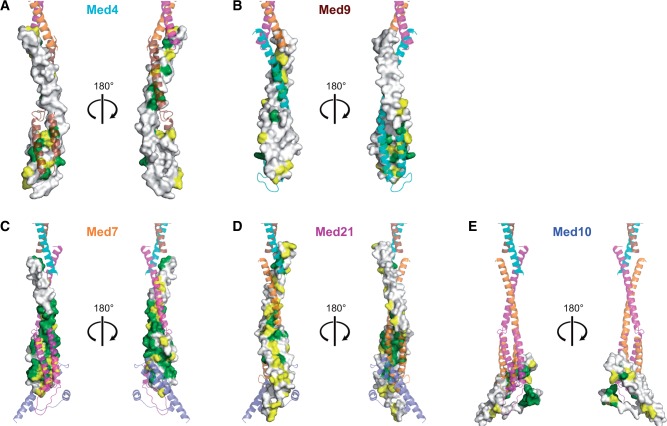
Conserved inter-subunit contacts within the model of the 6-subunit middle module. (**A**) Med4. (**B**) Med9. (**C**) Med7. (**D**) Med21. (**E**) Med10. Subunits are shown as surface representations. Residues are highlighted in green, yellow and white according to decreasing degree of conservation. Other subunits are represented as a semitransparent ribbon model colored as in Figure 1.

Here we derived a 3D architectural model of a 6-subunit Mediator middle module from yeast, which lacks only subunit Med1 (Figure 3, coordinate file provided as Supplementary Data S1). The model was obtained by combining available structural information with homology models, using as restraints experimental site-specific protein cross-linking data. The model comprises 744 residues of the module corresponding to a total of 69%. Of the 40 cross-linked residue pairs, 22 could be mapped onto the model and strongly support it. The other 18 cross-links map to the remaining 31% of the module residues that are predicted to be mainly flexible. These unstructured regions are generally dispensable for assembly of the module (Figure 2) (14). Our model comprises 82% of the 6-subunit middle module residues that were either shown or predicted to form secondary structure elements. Mapping conserved residues onto the model strongly supported the subunit arrangement and suggested that the architecture of the middle module is conserved over species, including human (Figure 7).

Our model is in agreement with reported data for the middle module. The length of the model is compatible with the previously determined hydrodynamic radius of the module (14). The elongated shape of the model is reminiscent of the recently published electron microscopic density of the endogenous middle module (28). All subunit interactions observed in the model have been reported using biochemical methods (13,14,29,30). There are only two described subunit interactions that the model does not account for, between Med10 and subunits Med4 and Med31 (14,29), but these interactions may be made by protein regions that could not be modeled (Figure 2). We previously reported that the complete 7-subunit middle module, which contains also Med1, is more compact than the 6-subunit module (14)*.* Because Med1 contacts several other subunits of the middle module (14,29), Med1 may cause a contraction of the module, but this could not be analyzed.

The most notable feature of our model is its length of ∼180 Å, which is consistent with the idea that Mediator stretches over a large surface area of Pol II. The model further emphasizes the flexible nature of the middle module, which explains why crystallization attempts were unsuccessful. The Med7C/Med21 subcomplex contains a flexible hinge between its 4-helix bundle domain and its coiled-coil protrusion (13). The Med4/Med9 heterodimer adopts a similar fold and likely harbors a similar hinge. This suggests that the terminal helix bundle domains in the 6-subunit middle module adopt their relative orientation and distance upon binding of Med1 or formation of higher-order complexes. Similarly, the head module contains several flexible domains. Different conformations of both modules may be expected when they can be trapped in higher-order complexes, such as an intact Mediator core complex or complexes with Pol II.

## SUPPLEMENTARY DATA

Supplementary Data are available at NAR Online.

## FUNDING

Boehringer Ingelheim fellowship and the Elite Network of Bavaria (to M.S.); Deutsche Forschungsgemeinschaft, [SFB646, TR5, GraKo1721, SFB960], CIPSM, NIM, an Advanced Grant of the European Research Council, the LMUinnovativ project Bioimaging Network, the Vallee Foundation and the Jung-Stiftung (to P.C.). Funding for open access charge: Deutsche Forschungsgemeinschaft.

*Conflict of interest statement*. None declared.

## Supplementary Material

Supplementary Data
