# Supplementary material for: Model of the Mediator middle module based on protein cross-linking
Source: Nucleic Acids Res. 2013 Aug 10;41(20):9266–73. doi: 10.1093/nar/gkt704 (PMC3814369; doi:10.1093/nar/gkt704)
Supplement: Supplementary Data [file supp_41_20_9266__index.html]

Model of the Mediator middle module based on protein cross-linking — Model of the Mediator middle module based on protein cross-linking — Supplementary Data 

# Model of the Mediator middle module based on protein cross-linking

## Supplementary Data

files

**Files in this Data Supplement:**

- Supplementary Data - txt file
- Supplementary Data - xls file
